# Supplementary material for: Learning Genetic Perturbation Effects with Variational Causal Inference
Source: bioRxiv. 2025 Jun 5:2025.06.05.657988. Preprint. [Version 1] doi: 10.1101/2025.06.05.657988 (PMC12157634; doi:10.1101/2025.06.05.657988)
Supplement: Supplement 1 [file NIHPP2025.06.05.657988v1-supplement-1.pdf]

bioRxiv preprint doi: https://doi.org/10.1101/2025.06.05.657988; this version posted June 5, 2025. The copyright holder for this preprint (which was not certified by peer review) is the author/funder, who has granted bioRxiv a license to display the preprint in perpetuity. It is made available under aCC-BY 4.0 International license.

A Supplementary Information

A.1 Code and Data Availability

Code and relevant data for the SCCVAE model and experiments are available at `https://github.com/uhlerlab/sccvae`.

A.1.1 Architecture and training.

The SCCVAE model is composed of the following:

1. *Expression encoder*: After the input cell expressions are log normalized, they are passed through 2 fully connected hidden layers of size 1024. Mean and variance encoders are fully connected layers of output size 512 that accept the output of the shared encoding. Each intermediate layer uses a leaky ReLU activation function with negative slope 0.2.

2. *Shift encoder*: 1 fully connected hidden layer of size 1024, followed by Leaky ReLU activation (negative slope=0.2) and an output layer of size 512. Output is softmaxed.

3. *SCM*: The causal graph is simulated using a size 512 × 512 parameter initialized to a Gaussian distribution with mean 0 and variance 0.1, multiplied by an upper triangular binary mask. Shift values are initialized as a 1 × 512 model parameter.

4. *Expression decoder*: 2 fully connected hidden layers of size 1024, followed by an output layer of size 8563. All layers except for output use LeakyReLU with negative slope 0.2.

The model was trained using a learning rate of 5e-4, a linear KL annealing schedule with $\beta_{max} = 1$, and MMD scale $\gamma = 10$. To represent the perturbation p, the top 50 PCA components were used. To stabilize training, a reconstruction term for the control distribution is added, where $U^{\varnothing}$ is decoded with $c^p = 0$ to reconstruct $X^{\varnothing}$. This term is scaled by 0.25 for in-distribution splits and out-of-distribution splits 0, 2, 3, and 4, and scaled by 1 for out-of-distribution split 1. Inclusion of this term reduces the risk of exploding gradients, but does not significantly affect numerical results.

A.1.2 Hyperparameter selection

Hyperparameter selection for learning rate, β, γ, and neural network size were performed using a grid search and selected based on lowest validation error according to the validation sets described in Section 3.1. Hyperparameters were selected from the following lists of possible values:

Learning Rate	[1e-3, 5e-4, 1e-4]
β	[1, 2, 4, 8]
γ	[1, 2, 4, 8, 10, 15]
X reconstruction term	[0.0, 0.25, 0.5, 0.75, 1.0]
Number of layers in encoder/decoder	[1, 2, 3]
Layer size	[512, 1024]

bioRxiv preprint doi: https://doi.org/10.1101/2025.06.05.657988; this version posted June 5, 2025. The copyright holder for this preprint (which was not certified by peer review) is the author/funder, who has granted bioRxiv a license to display the preprint in perpetuity. It is made available under aCC-BY 4.0 International license.

A.1.3 Shift selection.

After training, when presented with an unseen perturbation, we search for an optimal shift value within the range of c^p learned by the model. Using increments of 0.1, we grid search every possible shift value within range. For each shift value, we substitute for c^q and compute $\hat{X}^q$. The final value of c^q is set to the value that minimizes $MSE(X^q, \hat{X}^q)$, comparing to the mean expression value obtained from bulk data for perturbation q. Since we do not have access to an alterative bulk screen, we utilize pseudo-bulk expressions computed from the same dataset.

A.2 Quantitative Metrics

A.2.1 Mean Squared Error.

The mean squared error (MSE) metric evaluates the error between the means of the predicted and ground-truth distributions:

$$\text{MSE}(X^p, \hat{X}^p) = \mathbb{E}[(\mathbb{E}[X^p] - \mathbb{E}[\hat{X}^p])^2].$$

The final value reported is averaged across all genes of the mean normalized expression level.

A.2.2 Pearson Correlation.

We also evaluate the Pearson correlation between the distribution means, using a similar approach to the evaluation of MSE:

$$\text{PearsonR}(X^p, \hat{X}^p) = \frac{\text{cov}(\mathbb{E}[X^p], \mathbb{E}[\hat{X}^p])}{\sigma_{\mathbb{E}[X^p]}\sigma_{\mathbb{E}[\hat{X}^p]}}$$

where $\text{cov}(\cdot)$ is the covariance and $\sigma_{(\cdot)}$ is the standard deviation of each mean expression value.

A.2.3 Maximum Mean Discrepancy.

The maximum mean discrepancy (MMD) evaluates a distribution level error:

$$\text{MMD}(X^p, \hat{X}^p) = \|\mathbb{E}_{x^p \sim X^p}[\phi(x^p)] - \mathbb{E}_{\hat{x}^p \sim \hat{X}^p}[\phi(\hat{x}^p)]\|_2$$

where $\phi(\cdot)$ is the Gaussian kernel function.

A.2.4 Energy Distance.

To compute energy distance (D^2), we compare the prediction and ground truth on a distributional level as follows:

$$D^2(X^p, \hat{X}^p) = 2\mathbb{E}[d(X^p, \hat{X}^p)] - \mathbb{E}[d(X^p, X_1^p)] - \mathbb{E}[d(\hat{X}^p, \hat{X}_1^p)]$$

where X_1^p and $\hat{X}_1^p$ are independent samples from the same distributions as X^p and $\hat{X}^p$ respectively (implemented as random permutations of cells). The distance function is given by $d(X, Y) = \mathbb{E}[(X - Y)^2]$.

To correct for the effect of distribution sample size on error magnitude, MMD and Energy distance were computed in batches of size 32 averaged across the entire test dataset.

bioRxiv preprint doi: https://doi.org/10.1101/2025.06.05.657988; this version posted June 5, 2025. The copyright holder for this preprint (which was not certified by peer review) is the author/funder, who has granted bioRxiv a license to display the preprint in perpetuity. It is made available under aCC-BY 4.0 International license.

A.2.5 Direction of Changes.

We evaluate the quality of the model predictions on gene differential expression. For each perturbation, we compare the fraction of genes where the direction of change from the control cell distribution is the same as that of the ground truth (Fraction same), as well as the fraction of genes that are changed in the opposite direction (Fraction changed):

$$\begin{aligned} \Delta(X^p, \hat{X}^p) &= |(\mathbb{E}[X^p] - \mathbb{E}[X]) - (\mathbb{E}[\hat{X}^p] - \mathbb{E}[X])| \\ \text{Fraction\_same}(X^p, \hat{X}^p) &= Pr[\Delta(X^p, \hat{X}^p) = 0] \\ \text{Fraction\_changed}(X^p, \hat{X}^p) &= Pr[\Delta(X^p, \hat{X}^p) = 2] \end{aligned}$$

where X is the control control cell distribution.

A.3 Additional Results

A.3.1 Subset of perturbations.

Figure 7 shows the process for selecting perturbations for training. From the starting set of perturbations, approximately 20% are perturbations with 200 cells or more. Out of these, around 68% have a logistic regression score of 0.6 or more. Then, we remove perturbations that have no identified causal relationships with other observed genes, or are not included in the expression vector. This leaves 279 perturbational distributions for our analysis.

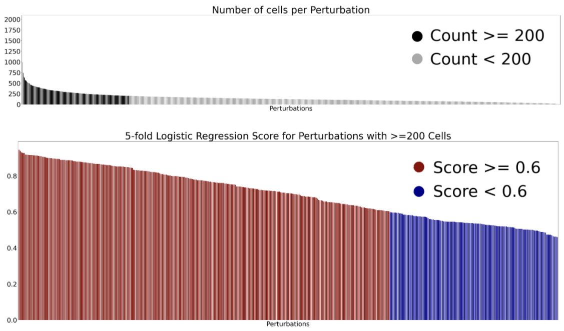

Figure 7: (A) We limit our analysis to perturbations with over 200 single cells. (B) Logistic regression filtering for perturbational distributions on the remaining perturbations. Only perturbations wtih a 5-fold cross validation logistic regression score of 0.6 or higher are considered.

A.3.2 In-distribution results.

Table 3 shows the results of SCCVAE training on the in-distribution data set. It can be seen that SCCVAE beats both GEARS and control baselines for all metrics, both on the entire set of essential genes and on the top 50 most highly variable genes in the control data set. In Table 4, we observe similar trends to the OOD results in that the original SCCVAE model outperforms the other versions of the model in MSE and MMD metrics. However, we note here that the Causal-GSP model does

bioRxiv preprint doi: https://doi.org/10.1101/2025.06.05.657988; this version posted June 5, 2025. The copyright holder for this preprint (which was not certified by peer review) is the author/funder, who has granted bioRxiv a license to display the preprint in perpetuity. It is made available under aCC-BY 4.0 International license.

not outperform the conditional model (except for energy distance), unlike in the OOD task, and the conditional model is also able to outperform the original SCCVAE on the energy distance metric. This is because the ability to utilize a causal graph and generalize to new cells is less crucial for the in-distribution task, since the test cells are from the same distribution as train cells.

**All genes**	**Control**	**GEARS**	**SCCVAE**
MSE	$0.0131_{\pm 0.00641}$	$0.0118_{\pm 0.00568}$	$\mathbf{0.0114_{\pm 0.004}}$
PearsonR	$\mathbf{0.982_{\pm 0.00841}}$	$\mathbf{0.982_{\pm 0.00833}}$	$\mathbf{0.982_{\pm 0.007}}$
MMD	$0.242_{\pm 0.0431}$	$0.239_{\pm 0.0440}$	$\mathbf{0.220_{\pm 0.01}}$
Energy Distance	$0.0363_{\pm 0.0200}$	$0.0325_{\pm 0.0176}$	$\mathbf{0.0296_{\pm 0.01}}$
Fraction Same	$0.497_{\pm 0.0127}$	$0.541_{\pm 0.0319}$	$\mathbf{0.587_{\pm 0.04}}$
Fraction Changed	$0.471_{\pm 0.0127}$	$0.459_{\pm 0.0319}$	$\mathbf{0.413_{\pm 0.04}}$

**Top 50 genes**	**Control**	**GEARS**	**SCCVAE**
MSE	$0.0153_{\pm 0.00970}$	$0.0134_{\pm 0.00846}$	$\mathbf{0.0126_{\pm 0.006}}$
PearsonR	$\mathbf{0.991_{\pm 0.00595}}$	$\mathbf{0.991_{\pm 0.00565}}$	$\mathbf{0.991_{\pm 0.004}}$
MMD	$0.249_{\pm 0.0602}$	$0.244_{\pm 0.0583}$	$\mathbf{0.227_{\pm 0.03}}$
Energy Distance	$0.0425_{\pm 0.0336}$	$0.0370_{\pm 0.0317}$	$\mathbf{0.0316_{\pm 0.02}}$
Fraction Same	$0.501_{\pm 0.0589}$	$0.548_{\pm 0.0762}$	$\mathbf{0.586_{\pm 0.08}}$
Fraction Changed	$0.459_{\pm 0.0589}$	$0.452_{\pm 0.0762}$	$\mathbf{0.414_{\pm 0.08}}$

Table 3: In-distribution results for SCCVAE compared to GEARS and the control baseline, for all essential genes and for top 50 highly variable genes, averaged across all perturbational distributions.

A.3.3 Breakdown of distance scatterplots by training/testing distributions.

Figure 8 looks at the correlation between MMD and latent space distance (similar to Figure 4) for train and test perturbations separately. There is a strong positive correlation in training test perturbations, as expected, but the test set perturbations also exhibit positive correlations across all five splits.

A.3.4 Additional shift selection results.

Figure 9 shows the same procedure as Figure 5 repeated on a different gene, NLE1. For NLE1, the post-perturbational expression distribution is much closer to the control distribution, and the shift value that yield the lowest error is also much smaller in magnitude ($c^p \approx 0.5$). We can see that as c^p become larger, the predicted distribution also moves farther away from the ground truth.

A.3.5 Additional perturbation modules.

Figure 10 visualizes the embeddings of 12 different functional perturbation modules in U^p latent space. As stated already, the embeddings of genes within one perturbational module form a cluster in the latent space. We observe that even across different perturbation modules affecting the same cellular

bioRxiv preprint doi: https://doi.org/10.1101/2025.06.05.657988; this version posted June 5, 2025. The copyright holder for this preprint (which was not certified by peer review) is the author/funder, who has granted bioRxiv a license to display the preprint in perpetuity. It is made available under aCC-BY 4.0 International license.

**All genes**	**SCCVAE**	**Conditional**	**Causal-GSP**	**Random**
MSE	**0.0114**$_{\pm\mathbf{0.00439}}$	0.0117$_{\pm 0.00473}$	0.0117$_{\pm 0.00426}$	0.0155$_{\pm 0.00396}$
PearsonR	**0.982**$_{\pm\mathbf{0.00742}}$	**0.982**$_{\pm\mathbf{0.00755}}$	0.981$_{\pm 0.00719}$	0.980$_{\pm 0.00708}$
MMD	**0.220**$_{\pm\mathbf{0.0180}}$	0.221$_{\pm 0.0220}$	0.221$_{\pm 0.0220}$	0.276$_{\pm 0.0234}$
Energy Dist.	0.0296$_{\pm 0.0183}$	0.0287$_{\pm 0.0182}$	**0.0266**$_{\pm\mathbf{0.0160}}$	0.0354$_{\pm 0.0110}$
Frac. Same	0.587$_{\pm 0.0475}$	0.575$_{\pm 0.0497}$	0.579$_{\pm 0.0473}$	**0.599**$_{\pm\mathbf{0.0385}}$
Frac. Changed	0.413$_{\pm 0.0476}$	0.425$_{\pm 0.0497}$	0.421$_{\pm 0.0473}$	**0.401**$_{\pm\mathbf{0.0385}}$

**Top 50 genes**	**SCCVAE**	**Conditional**	**Causal-GSP**	**Random**
MSE	**0.0126**$_{\pm\mathbf{0.00692}}$	0.0129$_{\pm 0.00692}$	0.0130$_{\pm 0.00687}$	0.0192$_{\pm 0.00643}$
PearsonR	**0.991**$_{\pm\mathbf{0.00487}}$	**0.991**$_{\pm\mathbf{0.00477}}$	**0.991**$_{\pm\mathbf{0.00495}}$	**0.991**$_{\pm\mathbf{0.00485}}$
MMD	**0.227**$_{\pm\mathbf{0.0339}}$	0.229$_{\pm 0.0360}$	0.228$_{\pm 0.0375}$	0.304$_{\pm 0.0400}$
Energy Dist.	0.0316$_{\pm 0.0293}$	**0.0312**$_{\pm\mathbf{0.0278}}$	0.0352$_{\pm 0.0270}$	0.0438$_{\pm 0.0203}$
Frac. Same	0.586$_{\pm 0.0815}$	0.577$_{\pm 0.0899}$	0.581$_{\pm 0.0813}$	**0.602**$_{\pm\mathbf{0.06}}$
Frac. Changed	0.414$_{\pm 0.0815}$	0.423$_{\pm 0.0899}$	0.419$_{\pm 0.0813}$	**0.398**$_{\pm\mathbf{0.06}}$

Table 4: Ablation studies on the in-distribution task. Unlike in the OOD task, the conditional and causal GSP models achieve better performance than the original SCCVAE for the energy distance metric.

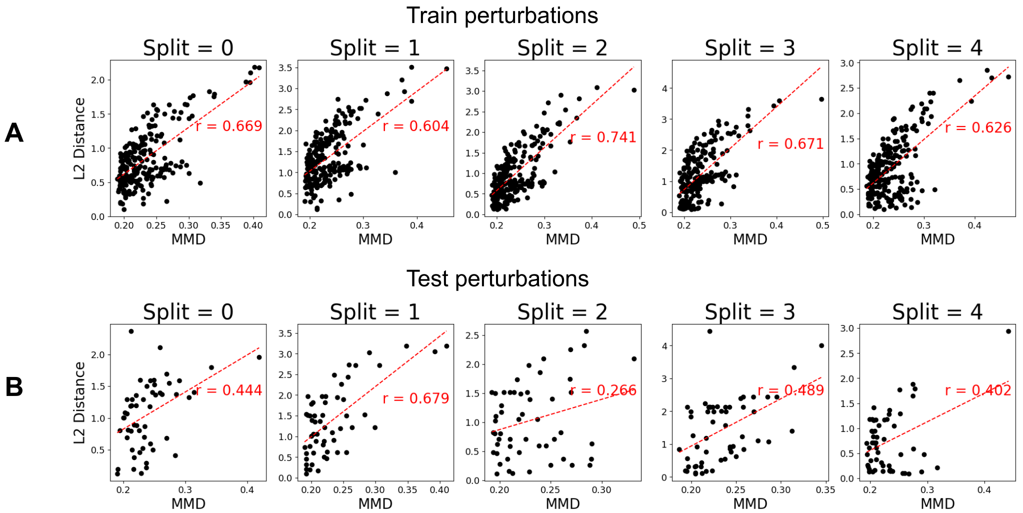

Figure 8: Continuation of Figure 4. (A) In just the training/validation set perturbations, the latent variables are learned directly during training (since no shift selection is required). This leads to stronger correlations overall. (B) In the test set, latent variables are computed after selecting shift to minimize MSE. Although the correlation is weaker than the training set perturbations, it is consistently positive across all splits.

bioRxiv preprint doi: https://doi.org/10.1101/2025.06.05.657988; this version posted June 5, 2025. The copyright holder for this preprint (which was not certified by peer review) is the author/funder, who has granted bioRxiv a license to display the preprint in perpetuity. It is made available under aCC-BY 4.0 International license.

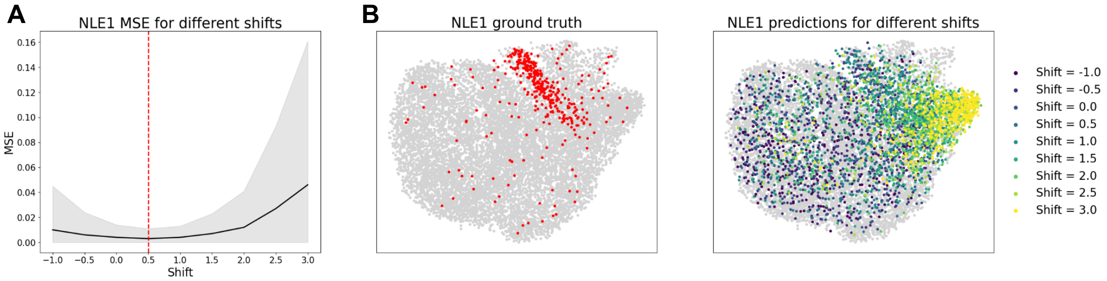

Figure 9: (A) Shift selection for a different perturbation (NLE1), where the optimal shift value is closer to control. (B) When c^p is increased, we can visualize the postperturbational cell expression distribution under a higher intensity gene knockdown.

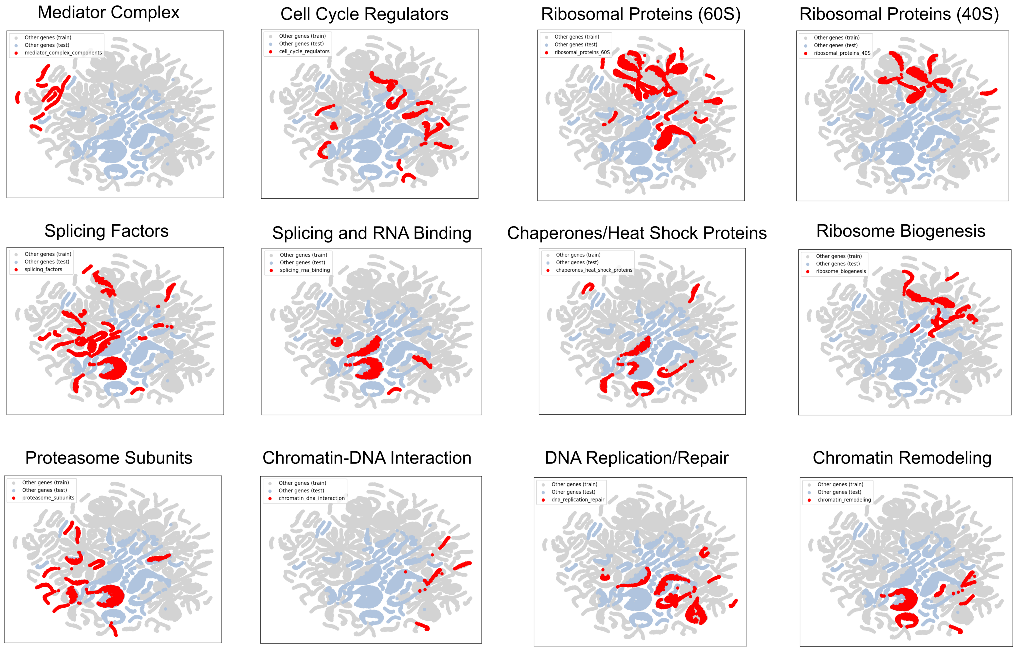

Figure 10: Individual perturbation modules. In each figure, the red regions indicate U^p embeddings of cells that are part of the perturbation module, the grey regions indicate U^p embeddings of non-module cells from the training set, and the blue regions indicate U^q embeddings of non-module cells from the test set.

functions, similar functional modules are also grouped closely in the latent space. For example, all genes encoding ribosomal proteins or ribosome biogenesis are close to each other, chromatin and DNA controlling genes are grouped closely together, and splicing genes are also grouped near each other.

bioRxiv preprint doi: https://doi.org/10.1101/2025.06.05.657988; this version posted June 5, 2025. The copyright holder for this preprint (which was not certified by peer review) is the author/funder, who has granted bioRxiv a license to display the preprint in perpetuity. It is made available under aCC-BY 4.0 International license.

A.3.6 Interpretation of the learned SCM.

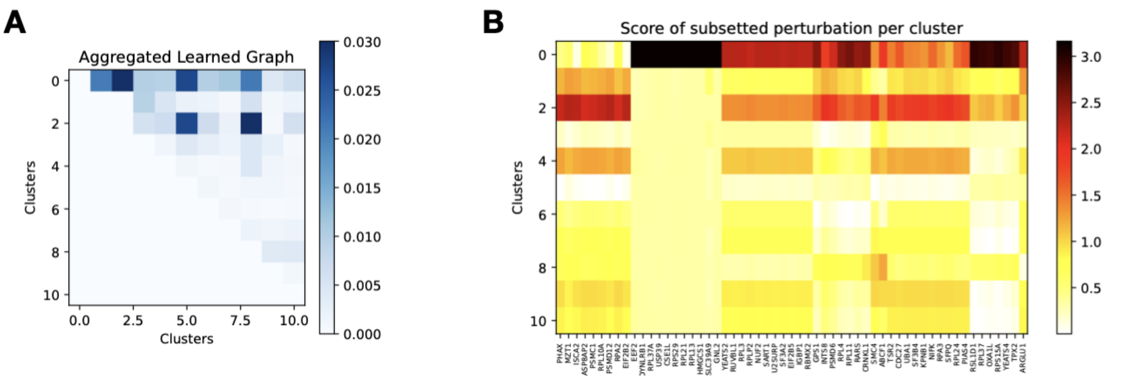

Figure 11: SCM Learned by SCCVAE. (A) Learned adjacency matrix A aggregated by clusters found using the encoded shift vectors S. (B) Learned shift vectors S averaged using the clusters found in (A) on a subset of perturbations.

Figure 11 visualizes the structural causal model learned by SCCVAE (Section 2.1) in the in-distribution experiment on $N = 279$ perturbations on K562 cells. As the learned graph $\mathcal{G}$ is encoded by the adjacency matrix $A \in \mathbb{R}^{512\times 512}$, we first use A to read out a learned network. The perturbations alter variables in this network by the encoded shift vectors $S = (S^{p_1}, \ldots, S^{p_{279}}) \in \mathbb{R}^{279\times 512}$.

To interpret the learned network, we cluster the dimensions of this network according to their responses to the perturbations. In particular, we use K-means clustering based on $S^\top$ with 11 clusters. We then aggregate the adjacency matrix A by averaging the scores between dimensions in each pair of clusters. A cluster is considered to be more upstream than a distinct cluster if in less than half of the entries in the sub-matrix of A corresponding these two clusters are zero. By re-orienting the clusters from upstream to downstream, we visualize the aggregated absolute connections in this network in Figure 11 (A).

To interpret how each perturbation S^p alters the individual clusters in the aggregated network, we average the entries of S^p corresponding to each cluster and use the averaged vector as the scoring for this perturbation. We cluster the perturbations based on the averaged scoring vector and visualize 10 random perturbations per cluster in Figure 11 (B). Notably cluster 0 is mainly contributed by the Ribosome family. A deeper understanding of these clusters may reveal biological insights.

A.3.7 Comparison across datasets.

We demonstrate the capacity of SCCVAE over other datasets. Out of the considered baselines, GEARS has shown to have consistent performances across metrics, therefore we compare against GEARS for analysis over other datasets. In Table 5, we compare SCCVAE to GEARS and control cell baselines on the entire dataset, without selecting for high signal-to-noise ratio perturbational distributions. Here, we observe that SCCVAE still generally out-performs baselines, although the difference is less dramatic than in the selected perturbations case. Additionally, both GEARS and control baselines appear to achieve lower error on the full dataset compared to the selected dataset,

bioRxiv preprint doi: https://doi.org/10.1101/2025.06.05.657988; this version posted June 5, 2025. The copyright holder for this preprint (which was not certified by peer review) is the author/funder, who has granted bioRxiv a license to display the preprint in perpetuity. It is made available under aCC-BY 4.0 International license.

whereas SCCVAE exhibits the opposite trend and achieves higher error on the full dataset. The most likely explanation for this is that the full dataset contains many perturbational distributions that are very similar to the control distribution. As a result, the control baselines are lower, and GEARS, which outputs similar predictions to control, achieves lower error. SCCVAE, which tends to identify differences from the control distribution, generally performs better when the perturbed distribution is distinct from the control distribution.

**All genes**	**Control**	**GEARS**	**SCCVAE**
MSE	$0.0077_{\pm 0.0077}$	$0.00731_{\pm 0.00745}$	$\mathbf{0.00657_{\pm 0.004}}$
PearsonR	$0.989_{\pm 0.0109}$	$0.989_{\pm 0.0107}$	$\mathbf{0.99_{\pm 0.006}}$
MMD	$0.213_{\pm 0.0286}$	$\mathbf{0.212_{\pm 0.0308}}$	$0.217_{\pm 0.02}$
Energy Distance	$0.0272_{\pm 0.0138}$	$0.0264_{\pm 0.0136}$	$\mathbf{0.0262_{\pm 0.01}}$
Fraction Same	$0.484_{\pm 0.0067}$	$0.522_{\pm 0.0226}$	$\mathbf{0.544_{\pm 0.04}}$
Fraction Changed	$0.483_{\pm 0.0067}$	$0.478_{\pm 0.0226}$	$\mathbf{0.456_{\pm 0.04}}$

**Top 50 genes**	**Control**	**GEARS**	**SCCVAE**
MSE	$0.00861_{\pm 0.00962}$	$0.00809_{\pm 0.0093}$	$\mathbf{0.00739_{\pm 0.006}}$
PearsonR	$\mathbf{0.995_{\pm 0.00643}}$	$\mathbf{0.995_{\pm 0.00634}}$	$\mathbf{0.995_{\pm 0.004}}$
MMD	$0.215_{\pm 0.0446}$	$\mathbf{0.214_{\pm 0.044}}$	$0.221_{\pm 0.03}$
Energy Distance	$0.0291_{\pm 0.0243}$	$\mathbf{0.0279_{\pm 0.0237}}$	$0.0283_{\pm 0.0}$
Fraction Same	$0.52_{\pm 0.0722}$	$0.528_{\pm 0.0744}$	$\mathbf{0.548_{\pm 0.08}}$
Fraction Changed	$0.48_{\pm 0.0722}$	$0.472_{\pm 0.0744}$	$\mathbf{0.452_{\pm 0.08}}$

Table 5: Evaluation on the entire K562 single-cell dataset, without selecting for high signal-to-noise ratio perturbations. Since GEARS exhibited the best performance out of the two model baselines, we evaluate against GEARS and the control baseline. SCCVAE achieves lower error than control or GEARS for all metrics except MMD with all essential genes and all metrics except MMD and Energy Distance with top 50 genes. Compared to evaluation on the selected perturbation dataset, evaluation on all genes appears to be an easier task for all models. This is likely due to the similarity of most distributions to control, as evidenced by the fact that GEARS exhibits a larger drop in performance on the selected dataset than SCCVAE.

Table 6 shows results upon evaluating a selected RPE1 dataset, also from Replogle et al. [31]. While SCCVAE continues to outperform GEARS and control cell baselines on most metrics when evaluating on all essential genes, both GEARS and SCCVAE consistently do not beat the control cell baseline when only the top 50 highly variable genes are considered, with SCCVAE usually achieving lower error than GEARS. There are two possible causes for this phenomenon. First, the RPE1 dataset is smaller than the K562 dataset, and the neural network based methods may require more data than is available to learn optimally. Second, the RPE1 cell line is non-cancerous, while the K562 cell line is cancerous, meaning that they derive from fundamentally different distributions. It is possible that these models are more performant on cancerous cell lines, as they may better capture the underlying biological variability and gene expression patterns that are more pronounced

bioRxiv preprint doi: https://doi.org/10.1101/2025.06.05.657988; this version posted June 5, 2025. The copyright holder for this preprint (which was not certified by peer review) is the author/funder, who has granted bioRxiv a license to display the preprint in perpetuity. It is made available under aCC-BY 4.0 International license.

in cancerous cells due to their altered regulatory mechanisms and higher levels of genetic instability. This suggests that these models might implicitly exploit features or patterns specific to cancer biology, leading to diminished performance on non-cancerous cell lines like RPE1, which exhibit more stable and less variable gene expression profiles. Future work could explore tailoring these models to account for differences in cell line characteristics or incorporating stronger priors to improve generalization across multiple different cell lines.

**All genes**	**Control**	**GEARS**	**SCCVAE**
MSE	$0.00715_{\pm 0.00782}$	$0.00635_{\pm 0.00704}$	$\mathbf{0.00634_{\pm 0.00618}}$
PearsonR	$0.988_{\pm 0.0127}$	$\mathbf{0.99_{\pm 0.0113}}$	$\mathbf{0.99_{\pm 0.00958}}$
MMD	$0.237_{\pm 0.0558}$	$\mathbf{0.233_{\pm 0.0511}}$	$0.239_{\pm 0.0446}$
Energy Distance	$0.0305_{\pm 0.0146}$	$0.03_{\pm 0.0142}$	$\mathbf{0.0299_{\pm 0.0134}}$
Fraction Same	$0.485_{\pm 0.00948}$	$0.503_{\pm 0.023}$	$\mathbf{0.545_{\pm 0.0524}}$
Fraction Changed	$0.485_{\pm 0.00948}$	$0.497_{\pm 0.023}$	$\mathbf{0.455_{\pm 0.0524}}$

**Top 50 genes**	**Control**	**GEARS**	**SCCVAE**
MSE	$\mathbf{0.0092_{\pm 0.0113}}$	$0.0122_{\pm 0.0253}$	$0.0121_{\pm 0.0238}$
PearsonR	$\mathbf{0.995_{\pm 0.00639}}$	$0.993_{\pm 0.0141}$	$0.994_{\pm 0.0132}$
MMD	$\mathbf{0.249_{\pm 0.074}}$	$0.265_{\pm 0.144}$	$0.277_{\pm 0.124}$
Energy Distance	$\mathbf{0.0343_{\pm 0.0248}}$	$0.0406_{\pm 0.0497}$	$0.04_{\pm 0.0498}$
Fraction Same	$0.485_{\pm 0.0756}$	$0.507_{\pm 0.075}$	$\mathbf{0.546_{\pm 0.0935}}$
Fraction Changed	$0.495_{\pm 0.0756}$	$0.493_{\pm 0.075}$	$\mathbf{0.454_{\pm 0.0935}}$

Table 6: Evaluation on the RPE1 cell line. SCCVAE achieves lower error than baselines when considering all essential genes, but the control cell baseline has lower error than both GEARS and SCCVAE when considering the top 50 highly variable genes.
